# Deep learning estimation of three-dimensional left atrial shape from two-chamber and four-chamber cardiac long axis views

**DOI:** 10.1093/ehjci/jead010

**Published:** 2023-02-02

**Authors:** Hao Xu, Steven E Williams, Michelle C Williams, David E Newby, Jonathan Taylor, Radhouene Neji, Karl P Kunze, Steven A Niederer, Alistair A Young

**Affiliations:** Department of Biomedical Engineering, King’s College London, Lambeth Palace Rd, London SE1 7EU, UK; Department of Biomedical Engineering, King’s College London, Lambeth Palace Rd, London SE1 7EU, UK; University/BHF Centre for Cardiovascular Science, University of Edinburgh, 47 Little France Crescent, Edinburgh EH16 4TJ, UK; University/BHF Centre for Cardiovascular Science, University of Edinburgh, 47 Little France Crescent, Edinburgh EH16 4TJ, UK; University/BHF Centre for Cardiovascular Science, University of Edinburgh, 47 Little France Crescent, Edinburgh EH16 4TJ, UK; 3DLab, Sheffield Teaching Hospitals NHS Foundation Trust, Northern General Hospital, Sheffield, s5 7AU, UK; Department of Biomedical Engineering, King’s College London, Lambeth Palace Rd, London SE1 7EU, UK; MR Research Collaborations, Siemens Healthcare Limited, Newton House, Sir William Siemens Square, Frimley, Camberley, Surrey, GU16 8QD, UK; Department of Biomedical Engineering, King’s College London, Lambeth Palace Rd, London SE1 7EU, UK; MR Research Collaborations, Siemens Healthcare Limited, Newton House, Sir William Siemens Square, Frimley, Camberley, Surrey, GU16 8QD, UK; Department of Biomedical Engineering, King’s College London, Lambeth Palace Rd, London SE1 7EU, UK; Department of Biomedical Engineering, King’s College London, Lambeth Palace Rd, London SE1 7EU, UK

**Keywords:** left atrial volume, machine learning, cardiovascular magnetic resonance

## Abstract

**Aims:**

Left atrial volume is commonly estimated using the bi-plane area-length method from two-chamber (2CH) and four-chamber (4CH) long axes views. However, this can be inaccurate due to a violation of geometric assumptions. We aimed to develop a deep learning neural network to infer 3D left atrial shape, volume and surface area from 2CH and 4CH views.

**Methods and results:**

A 3D UNet was trained and tested using 2CH and 4CH segmentations generated from 3D coronary computed tomography angiography (CCTA) segmentations (*n* = 1700, with 1400/100/200 cases for training/validating/testing). An independent test dataset from another institution was also evaluated, using cardiac magnetic resonance (CMR) 2CH and 4CH segmentations as input and 3D CCTA segmentations as the ground truth (*n* = 20). For the 200 test cases generated from CCTA, the network achieved a mean Dice score value of 93.7%, showing excellent 3D shape reconstruction from two views compared with the 3D segmentation Dice of 97.4%. The network also showed significantly lower mean absolute error values of 3.5 mL/4.9 cm^2^ for LA volume/surface area respectively compared to the area-length method errors of 13.0 mL/34.1 cm^2^ respectively (*P* < 0.05 for both). For the independent CMR test set, the network achieved accurate 3D shape estimation (mean Dice score value of 87.4%), and a mean absolute error values of 6.0 mL/5.7 cm^2^ for left atrial volume/surface area respectively, significantly less than the area-length method errors of 14.2 mL/19.3 cm^2^ respectively (*P* < 0.05 for both).

**Conclusions:**

Compared to the bi-plane area-length method, the network showed higher accuracy and robustness for both volume and surface area.

## Graphical Abstract

**Graphical Abstract:**
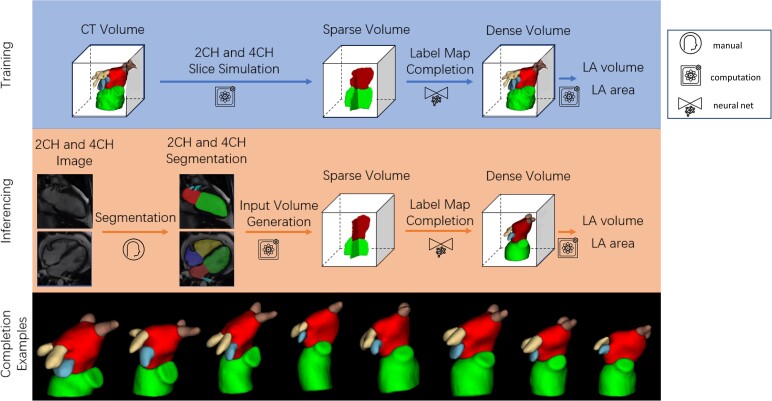

## Introduction

Left atrial (LA) volume is an important prognostic indicator of adverse events in patients with cardiovascular disease, including atrial fibrillation^1^ and heart failure.^2^ In addition, LA surface area^3^ and shape^4^ may provide additional prognostic value. However, volumetric data are often unavailable, due to scan time and resource constraints. LA volumes are commonly estimated using the bi-plane area-length method from two-chamber (2CH) and four-chamber (4CH) long axis cine images which are routinely acquired with echocardiography or cardiovascular magnetic resonance (CMR).^1,5,6^ Due to the limitations of the geometric assumptions (including a regular ellipsoidal LA shape), the bi-plane area-length method may lead to large volume errors, and surface area and shape are not well characterized. A more robust and accurate method for estimating LA volume and shape would enable better characterization of patients from routinely acquired 2CH and 4CH views, identify which patients may benefit from additional investigation (e.g. volumetric atrial imaging), and more precisely evaluate longitudinal changes from previous scans.

Recent advances in convolutional neural networks show promise in reconstructing 3D shapes from sparse or incomplete input data through label-to-label mapping.^7,8^ We sought to determine whether 3D LA shape reconstruction from 2CH and 4CH cine views would enable a more accurate estimation of LA volume and surface area. Automated analysis of LA 3D volume from standard long axis planes would enable in-line application at the time of scanning and highlight which patients may benefit from further examination.

In this study, we developed a deep learning algorithm to accurately estimate LA volume and surface area by reconstructing the 3D shape of the LA from 2CH and 4CH image segmentations. Since the method was designed to work from 2CH and 4CH segmentations, independent from image contrast, we trained and validated our method using 2CH and 4CH segmentations generated from 3D coronary computed tomography angiography (CCTA) exams, in locations matching CMR view orientations and allowing for motion between the two scans due to differences in breath-hold positions. This approach enables the network to learn the underlying 3D geometry from the sparse 2CH and 4CH information, and to correct for motion between scans, since the ground truth is known exactly. We then tested the method in an external test dataset comprising CMR 2CH and 4CH acquisitions, against paired CCTA acquisitions. We hypothesized that the network would be able to estimate LA volume more accurately than area-length methods, and would also be able to estimate LA surface area.

## Methods

**Figure 1:**
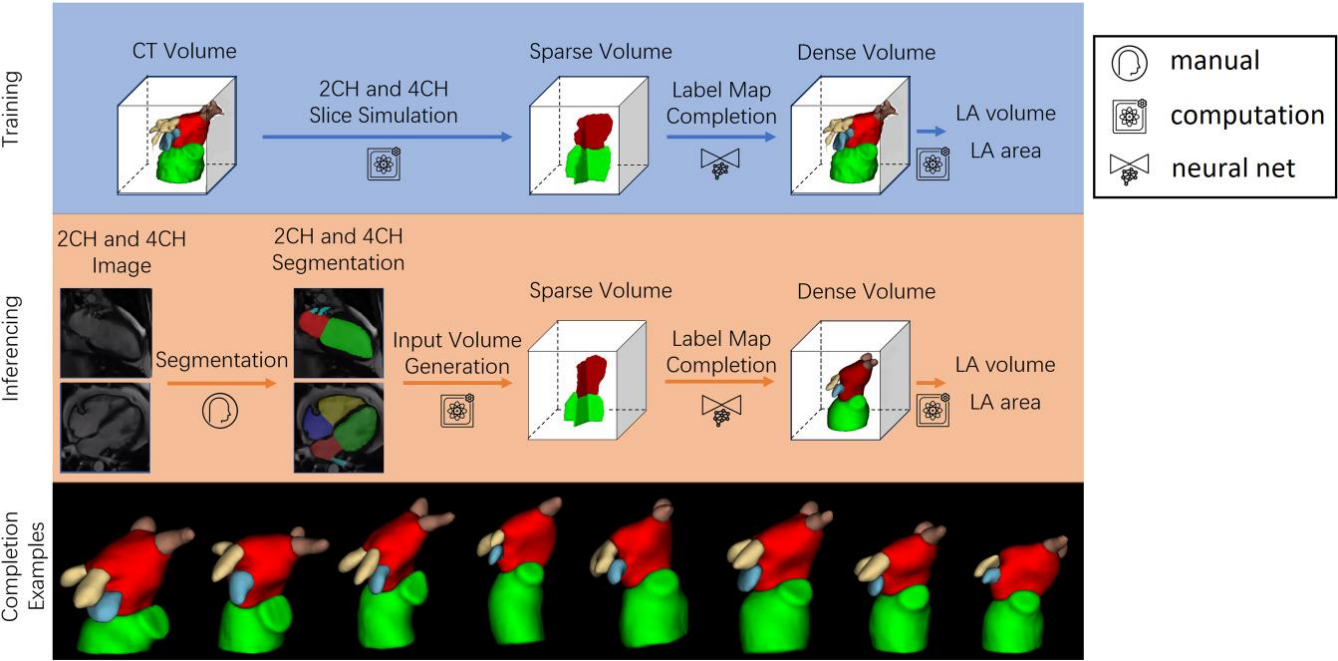
Overall pipeline. The green, red, blue, yellow and brown labels are the LV, LA, LA appendage, left pulmonary vein and right pulmonary vein respectively. Top: Training pipeline using CCTA. Middle: Inferencing pipeline using CMR. Bottom: examples of a variety of network outputs.

In this retrospective study, we trained and tested the network using accurate ground truth 3D segmentations obtained from CCTA in 1700 patients with suspected coronary artery disease, by simulating 2CH and 4CH slice segmentations together with random errors in slice and breath-hold positioning (motion artefact common in CMR). We also tested the method in an independent cohort of 20 patients with paired CMR and CCTA exams, using the CMR 2CH and 4CH segmentations as input and the 3D CCTA as the ground truth. *Figure 1* shows the overview of our method, including the training and inferencing stages, with examples of a variety of predicted LA shapes obtained from the network.

The training stage consisted of three steps. Firstly, 3D CCTA images were segmented with LV, LA, LA appendage, left pulmonary vein, and right pulmonary vein labels. Secondly, 2CH and 4CH label maps with only LV and LA labels were generated from segmented CCTA images, using an online augmentation process that added random offsets in 2CH/4CH orientations and positions, consistent with slice planning variation and breath-hold misregistration artefacts common in CMR, for each epoch of the training stage. The labels were then transferred to a normalized 3D space defined by the position and orientation of the augmented 2CH and 4CH planes. Thirdly, the sparse input label map volume was converted to a dense label map by the label completion network, giving dense volumetric label maps of the LA, LV, left/right pulmonary veins, and LA appendage.

Similarly, the inference stage consisted of four steps. Firstly, the 2CH and 4CH CMR images were segmented, giving 2D LV and LA blood pool label maps for each view. The 2D label maps were then transferred to the normalized 3D space. The sparse input label map volume was then converted to a dense label map by the label completion network, giving dense volumetric label maps of the LA, LV, left/right pulmonary veins, and LA appendage. Finally, the LA volume was calculated from the dense label maps with voxel summation, and the LA surface area was evaluated from a marching cube surface representation computed from the dense label map. Since the method works on segmentations, not original images, the same inferencing workflow can be applied to test datasets generated from both CCTA and CMR acquisitions.

The following sections describe each stage in more detail.

### Datasets

All data were anonymized in accordance with GDPR protocols prior to the study. The network was trained and validated using 3D LA shapes obtained from 1700 segmented CCTA images (label maps) of patients who participated in the Scottish Computed Tomography of the Heart (SCOT-HEART) trial.^9^ Briefly, all patients had suspected angina attributable to coronary artery disease and were imaged between 2010 and 2014 at one of three sites using either 64- or 320-detector row scanners (Brilliance 64, Philips Medical Systems, Netherlands; Biograph mCT, Siemens, Germany; Aquilion ONE, Toshiba Medical Systems, Japan). Tube current, voltage, and volume of iodine-based contrast were adjusted based on body mass index.^10^ Of the 1768 SCOT-HEART cases with adequate CCTA, 68 cases were removed due to poor segmentations from the automated segmentation process.^10^

An independent cohort of 20 cases from an external site (not part of SCOT-HEART) was also used to evaluate the method. CMR 2CH and 4CH image segmentations were used as input and 3D CCTA segmentations were used as the ground truth. Similar to the SCOT-HEART cohort, these patients had suspected angina attributable to coronary artery disease. They were imaged between 2017 and 2020 with an average of 2.5 months between CCTA and CMR scans. The CT scanners were Aquilion ONE, Toshiba Medical Systems, Japan, and the MR scanners were Aera and Avanto-fit, Siemens, Germany.

### CCTA training and testing data

The CCTA images were segmented automatically using a previously described and validated 3D convolutional neural network.^10^ This provided ten labels, including LA, LV, RV, left/right pulmonary veins, and LA appendage, with a Dice score of 97.4% for the LA compared with manual segmentations.^10^ The 3D segmentations were reviewed visually to ensure high quality. We generated 2CH and 4CH long axis views from the 3D label maps, in the same orientations acquired in CMR exams, including the potential for patient motion between the two scans, as follows.

**Figure 2:**
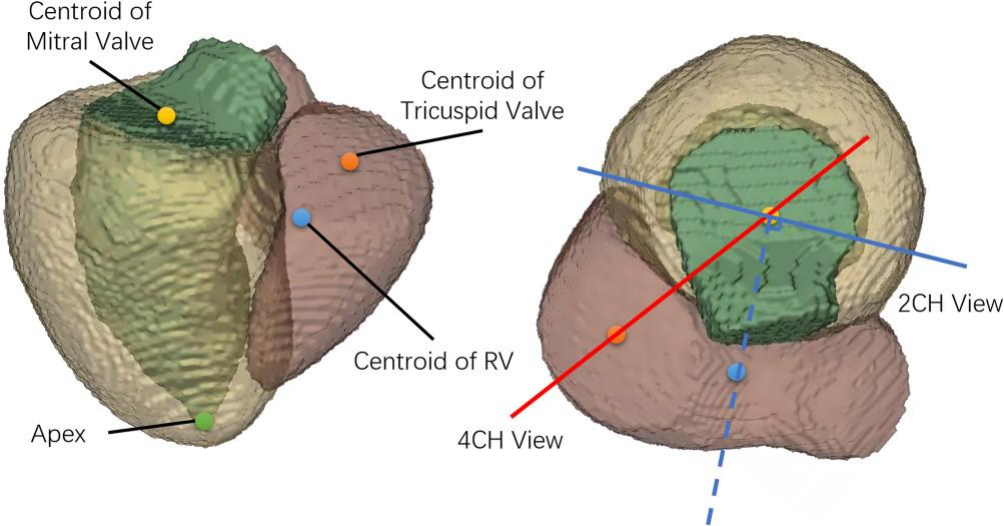
Definition of the 2CH and 4CH views. The green, yellow, and pink segments are LV, LV myocardium and RV. The yellow, orange, blue and green points are the centroid of mitral valve, the centroid of tricuspid valve, the centroid of RV and the apex. The solid red and blue lines indicate the 4CH and 2CH views visualized along the long axis. The solid blue line is perpendicular to the dotted blue line passing through the centroids of RV and mitral valve.

Firstly, the plane passing through the centroid of the mitral valve, the centroid of the tricuspid valve, and the LV apex, was defined as the 4CH view. Secondly, we calculated the centroid of the right ventricle (RV), and the plane that passes through the centroid of mitral valve, the apical point, and the plane normal in the direction of the centroid of the RV was defined as the 2CH view. The long axis was the intersecting line of the two views, which passes through the centroid of mitral valve and the apical point. A normalized 3D LA-centric coordinate system was defined using the long axis, the normal vector of the 4CH view (front-to-back), and the cross product of the two (left-to-right), as axes, taking the centroid of the LA as the origin. The definitions of the 2CH and 4CH views are shown in *Figure 2*. A volume with voxel spacing of 1 mm^3^ and the size of 128^3^ was generated based on the LA coordinates and centred at the origin. The five labels of interest (LA, LV, left/right pulmonary veins, and LA appendage) were then resampled to the normalized coordinates and considered as the reference dense volume. When preparing the dense volume, we also introduced errors in the origin and long axis orientation to simulate variation in real MR slice planning, by applying a random translation offset within ±10 pixels along each axis, a random rotation within ±10°around each axis.

To generate the sparse input volume, we first prepared a binary mask for each of the long axis planes by labelling voxels further than 1 mm from the plane as 0 (background). A random rotation offset within ±15°of the 2CH view around the long axis was applied to augment the angle between 2CH and 4CH views, simulating variation in real MR slice planning. We also simulated the effect of breath-hold motion artefacts when generating the input sparse volume. We added a Gaussian distributed random offset with a mean of 1.6 mm and standard variance of 0.62 mm along each axis, estimated from measured breath-hold motions within the UK Biobank dataset,^11^ to the label map volume before applying voxel-wise multiplication between the binary mask and misaligned volume. The 2CH and 4CH sparse volumes are finally fused to form the input sparse volume, and the intersecting voxels take labels from the 2CH view. Since only the LV and LA labels are commonly segmented for 2CH and 4CH CMR images, we only kept these two labels and removed others from the input volume. Typical sparse volume examples are shown in *Figure 1*.

We used 1400 cases to train the network parameters, and 100 cases for validation of the optimized network parameters (to monitor overfitting). The remaining 200 cases were used to test the performance of the network, compared to 3D ground truth and the area-length method. The split was performed randomly, with approximately the same demographic and disease proportions in each group. The same offset process, simulating breath-hold motion and positioning errors, was applied to the 200 testing cases to generate input sparse volumes, in order to evaluate the performance of the label completion network.

### Paired CMR and CCTA test data

The LV and LA labels were first segmented manually from the CMR images using ITK-SNAP (itksnap.org), and transformed into the reference sparse 3D volume in the same way as above. CMR images consisted of multiple frames and the frame with the closest time to the CCTA scan (relative to the R wave) was chosen to build the input sparse volume. The paired CCTA images were segmented automatically using the previously described segmentation network .^10^

### LA volume and surface area

The bi-plane area-length method^12^ calculates the LA volume using the areas of the LA and lengths of the LA (usually defined as the distance from the mitral valve to the furthest point of LA) from 2CH and 4CH images:


$$LA\;volume=\;\frac{8\times A_{2CH}\times A_{4CH}}{3\pi L}$$

where A_2CH_ and A_4CH_ are the areas of the LA calculated from 2D segmentations, and L is the length of LA. The geometric assumptions of the area-length method include: (1) the LA shape is an ellipsoid; (2) the 2CH and 4CH are perpendicular; (3) the LA length is along the long axis. In this study we compared two definitions of the LA length, and for each definition we used the shorter length, mean value, and longer length of the two views to calculate the volume. The traditional definition calculates the lengths independently for each view and measures the distance from the mitral valve to the furthest point of LA. This was widely applied in echocardiography as the long axis is not available, and then was adapted to CMR images. We also tested a modified definition of the LA length as the intersecting distance along the long axis of the two views, taking the advantage that the CMR imaging planes are known.

We calculated a LA surface area based on the same geometric assumptions, using principal semiaxes calculated from A_2CH_, A_4CH_, and L:


$$a=\frac{A_{2CH}}{\pi L}$$

$$b=\frac{A_{4CH}}{\pi L}$$

The surface area of the LA was then estimated using Knud Thomsen's formula with *p* = 1.6 as:


$$LA\;area=4\pi \times {\left(\frac{{\left(a\times b\right)}^{1.6}+{\left(a\times L\right)}^{1.6}+{\left(b\times L\right)}^{1.6}}{3}\right)}^{1/1.6}$$

For volumetric data, we calculated the volume by summing the voxels with the LA label and multiply by the corresponding voxel volume, which is 1 mm^3^. We calculated the mesh surface area generated from the LA label map using the marching cubes algorithm, after the surface was first passed through a 3D Lanczos kernel to filter out the high-frequency noise introduced through interpolation and resampling.

The estimated surface area from the area-length method includes the entire LA. However, intersections with the LV (i.e. mitral valve area), pulmonary veins, and LA appendage will reduce this area estimate. In atrial fibrillation patients, this reduced area estimate is likely to be more informative since it contains only the LA muscle wall area.^3^ Therefore, we calculated two different types of LA areas from the dense label maps. The first one is the total LA surface area, corresponding to the area-length method. The second one removed the intersections between the LA and other labels, and this area measures the surface of the atrial wall only. This was done by first finding the voxels touching voxels with other labels (e.g. mitral valve, LA appendage, and pulmonary veins) and removing these from the marching cubes surface area calculation.

### Label completion network design and training

A 3D U-Net^13^ was implemented as the label completion network using pytorch 1.81. This was designed to take a sparse label input consisting of three labels (background (0), LA (1), and LV (2)), and to output a dense label map consisting of five labels (background (0), LA (1), LV (2), left pulmonary veins (3), right pulmonary vein (4), and LA appendage (5)). The network had four spatial resolutions, and the convolutional kernels had the size of 3 × 3 × 3 with the number of (16, 32), (32, 64), (64, 128), and (128, 256) in the encoder and bottle neck, and (128, 128), (64, 64), (32, 5) in the decoder. Max-pooling and deconvolution with a stride of 2 × 2 × 2 were used for contraction and expansion. The Adam optimization^10^ with a learning rate of 10^−3^ was used for training and Dice score of the LA was used for training and evaluation of the network. Weight initialization was random using default PyTorch settings for individual layers. Augmentation was performed during each epoch to create a larger training set.

### LA shape

The Dice metric was used to evaluate differences in LA shape. This measures the overlap in voxels between the prediction and the ground truth as a percentage of the object size. The largest connected component of the LA label was first extracted from the output of the network before the volume and Dice score were calculated. To correct for offsets between the output LA shape and the ground truth LA shape due to breath-hold misregistration, a rigid alignment^14^ was applied to map the prediction to the ground truth (i.e. translation and rotation). The rigid alignment was performed by minimizing the differences between signed distance maps computed from both segmentations, applying rotations and translations to one shape to match the other. This was applied to the predicted LA label map before the calculation of the Dice score, to ensure that the Dice metric was sensitive to changes in shape only.

### Statistics

Absolute errors in volume and surface area were compared between the network and bi-plane area-length method using a paired *t*-test. Differences in Dice score between patient sub-groups were compared using unpaired t-tests. Normality was tested using the Chi Square test at a 95% confidence level. Non-normal data were compared using the Mann–Whitney U test. A *P* value of 0.05 was considered significant.

## Results

### Study population characteristics

The study population characteristics are summarised in *Table 1*. The statistics of the LA sizes from CCTA for the two test cohorts, i.e. 20 test cases with paired CMR images and the 200 test cases with CCTA images are summarized in *Table 2*.

**Table 1:** Study population characteristics. Values are presented as mean ± standard deviation or (% with respect to number of total number of cases)

	CCTA *n* = 1700	CMR + CCTA *n* = 20
Age (years)	57.6 ± 9.5	62.0 ± 8.7
Male	955 (56%)	11 (55%)
Weight (kg)	85.4 ± 17.7	84.5 ± 17.3
Height (m)	1.70 ± 0.10	1.68 ± 0.08
BMI (kg/m^2^)	29.6 ± 5.5	29.7 ± 4.8
AF	30 (2%)	NA
Diabetes	185 (11%)	NA
CHD	166 (10%)	10 (50%)
Hypertension	582 (34%)	NA

AF, atrial fibrillation; BMI, body mass index; CHD, coronary heart disease. NA, not available.

**Table 2:** LA size distributions (from CCTA)

	LA volume (ml)	Total LA area (cm^2^)	LA wall area (cm^2^)
CMR + CCTA *n* = 20	92.5 ± 30.8	115.3 ± 20.8	81.5 ± 17.1
CCTA Test *n* = 200	82.3 ± 23.5	121.8 ± 24.2	88.6 ± 18.9

Values are presented as mean ± standard deviation.

### CCTA test cases

On the 200 randomly selected test cases, the Dice score of the LA had a mean value of 93.7%, a standard deviation of 1.7%, and an inter-quartile range of 1.9%. This result implies excellent 3D shape recovery from two views compared with the 3D shape obtained with the 3D segmentation CNN. In order to test if disease sub-groups had any impact on the shape reconstruction accuracy, we performed t-tests for differences in Dice between sub-groups of sex, atrial fibrillation, diabetes, coronary heart disease, and hypertension history and found no significant differences between any of the groups (*Table 3*).

**Table 3:** Dice scores and test results of groups with/without AF, male/female, with/without diabetes, with/without CHD, and with/without hypertension (*n* = 200 test cases from CCTA cohort)

	Group	Dice score (%)	*P*-value
AF	Yes (*n* = 5)	94.3 ± 1.2	0.42
	No (*n* = 195)	93.7 ± 1.8	
Sex	Male (*n* = 123)	93.7 ± 1.9	0.90
	Female (*n* = 77)	93.7 ± 1.5	
Diabetes	Yes (*n* = 26)	94.1 ± 1.2	0.24
	No (*n* = 174)	93.7 ± 1.8	
CHD	Yes (*n* = 20)	94.0 ± 2.0	0.52
	No (*n* = 180)	93.7 ± 1.7	
Hypertension	Yes (*n* = 65)	94.0 ± 1.5	0.08
	No (*n* = 133)	93.6 ± 1.8	

Dice score values are presented as mean ± standard deviation.

AF, atrial fibrillation; CHD, coronary heart disease.

The absolute and signed errors of the modified area-length method and network reconstruction estimations are shown in *Table 4*. The selection of the best area-length method is detailed in the *Supplementary Materials*, and our modified area-length method was the most accurate and robust out of all the variations tested.

**Table 4:** LA size estimation accuracy (*n* = 200 test cases generated from CCTA)

		LA volume (ml)	Total LA area (cm^2^)	LA wall area (cm^2^)
Area-length method	AE	13.0 ± 12.5	34.1 ± 50.4	NA
	SE	7.3 ± 16.4	20.7 ± 57.2	NA
Network reconstruction	AE	3.5 ± 3.2*	4.9 ± 3.6*	4.2 ± 2.9
	SE	−0.8 ± 4.6	4.1 ± 4.5	−3.2 ± 4.0

Values are presented as mean ± standard deviation.

AE, absolute error; SE, signed error. NA, not applicable.

*P* < 0.05 network vs. area-length method.

The network estimated the total LA volume with a nearly three-fold reduction in mean absolute error relative to the area-length method (*P* < 0.05), and reduced the total surface area mean absolute error by nearly seven-fold (*P* < 0.05).

### External CMR validation

The Dice score of the LA had a mean value of 87.4%, a standard deviation of 3.4%, and an inter-quartile range of 3.3%. The absolute and signed errors between the network reconstructed LA size from real CMR segmentations and the reference LA size calculated from paired CCTA segmentations of the 20 cases used for validation are shown in *Table 5*.

**Table 5:** LA size estimation accuracy *n* = 20 external CMR dataset

		LA volume (ml)	LA area (total) (cm2)	LA area (wall) (cm2)
Area-length method	AE	14.2 ± 10.9	19.3 ± 19.6	NA
	SE	−11.3 ± 14.0	2.1 ± 27.8	NA
Network reconstruction	AE	6.0 ± 6.3*	5.7 ± 4.1*	7.2 ± 5.1
	SE	−0.2 ± 8.8	0.9 ± 7.1	−6.5 ± 6.0

Values are presented as mean (standard deviation).

AE, absolute error; SE, signed error.

*P* < 0.05 network vs. area-length method.

The network estimated the total LA volume with over two-fold reduction in mean absolute error relative to the area-length method (*P* < 0.05), and the reduced total surface area mean absolute error by three-fold (*P* < 0.05).

**Figure 3:**
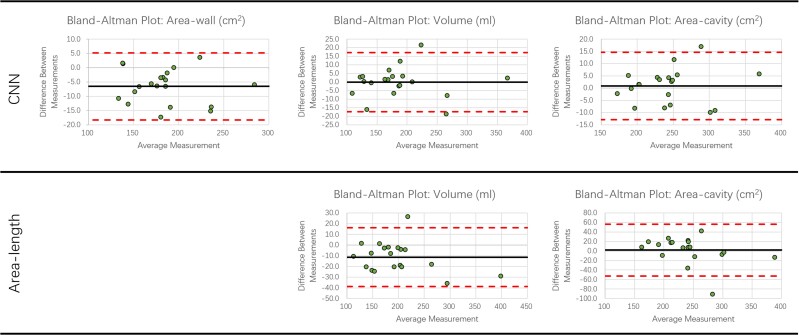
Bland-Altman plots. Plots show errors for the LA volume, LA wall area and total LA area for network and area-length methods (CMR completion vs. CCTA ground truth). The black line indicates the mean difference between measurements. The upper and lower dashed red lines indicate the ±1.96 standard deviation of the difference between measurements away from the mean value.

Bland-Altman plots of the LA volume, total LA area, and LA wall area for both network and area-length methods, compared with 3D ground truth, are shown in *Figure 3*. The differences between the measurements were calculated by subtracting the predicted values from the reference values. The bias for the network was small, with the largest mean bias shown for the LA wall area of 6.5 cm^2^. The limits of agreement were substantially reduced for the network compared with the area-length method.

**Figure 4:**
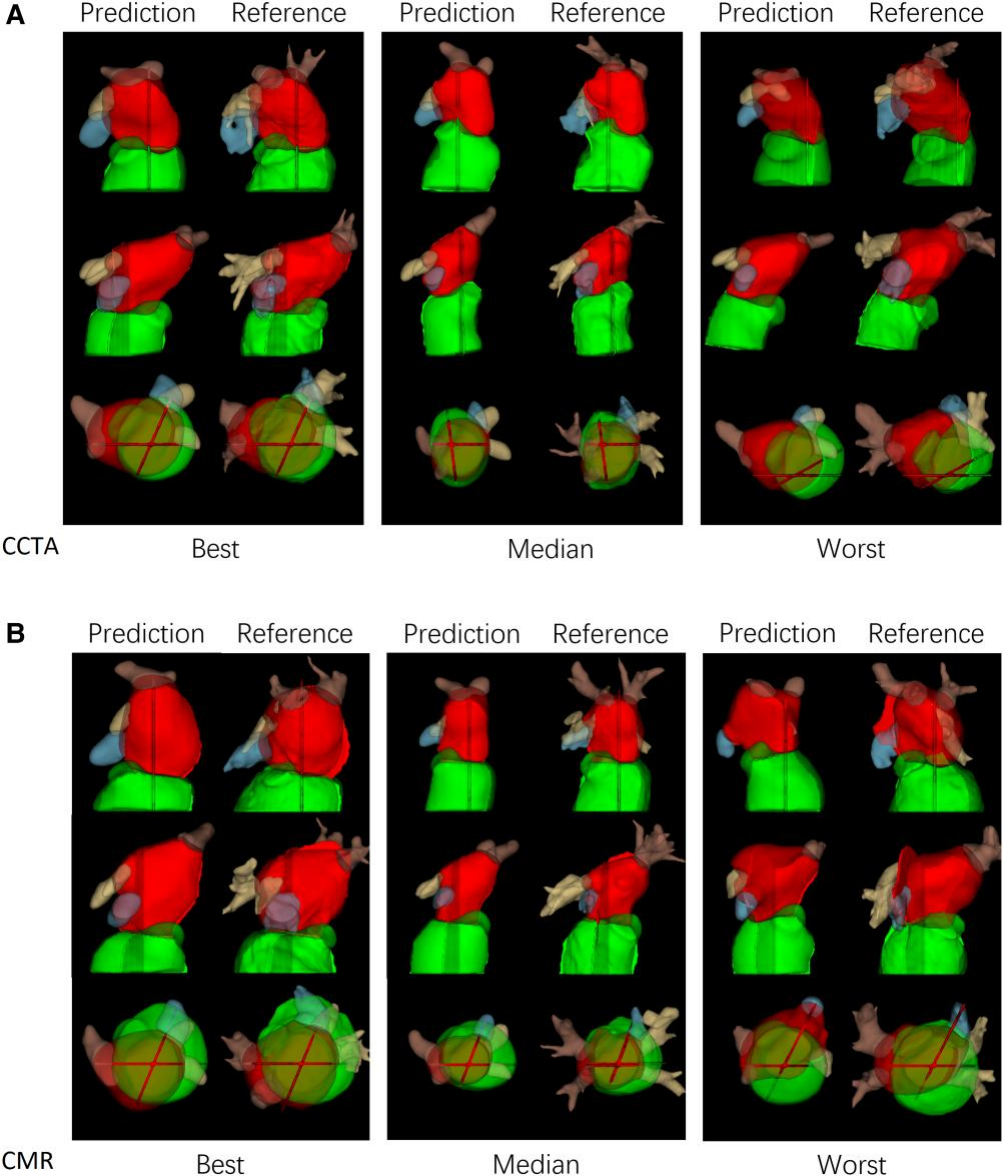
(*A*) Best, median and worst-case examples from CCTA test cases. (*B*) Best, median and worst-case examples from CMR test cases.

*Figure 4* illustrates the best, median, and worst cases for the CCTA and CMR test sets. In general, performance dropped if the 4CH and 2CH slices did not pass through the centre of the LA due to either slice positioning error, or tilting of the LA relative to the long axis of the LV.

## Discussion

In this study, we have developed an automated method for LA assessment from standard 2CH and 4CH views, which are typically acquired in all CMR exams. This additional information will add value to standard CMR protocols by providing more accurate and robust LA volume, surface area, and shape from standard views. This estimate could be used to rapidly flag cases in which a more detailed assessment is indicated, e.g. including atrial fibrosis mapping. Alternatively, the method could be used to efficiently monitor the effects of treatment on LA size and shape.

### Area-length method

Unlike echocardiography, the position and orientation of CMR images are known in patient coordinates. We proposed a LA length definition based on the long axis for CMR images, and the accuracy and robustness of the area-length method were improved as it was more robust to errors in slice positioning than the standard method of estimating the length independently from 2CH and 4CH views. However, the performance of the area-length method was limited by the geometric assumptions. *Figure 1* illustrates the large variation of the LA shapes, and the geometric assumption that LA is an ellipsoid is not strongly supported. By assuming the long axis aligns with one of the semi-axes, and that each of the 2CH and 4CH views contain another semi-axis of the ellipsoid, the two views are assumed to be perpendicular to each other, which may not be valid for typical CMR images.

### 3D Label map completion

The LA shape reconstruction network was much more accurate for both generated CCTA and external CMR test cases compared to the area-length method, and both the bias (signed difference) and absolute error of the estimated LA size were dramatically reduced. The key advantage of our deep learning method, which is also the main reason for the better performance, is that it has no geometric assumptions and adapts to a larger variation of the LA shapes while being robust to errors in the position of the 2CH and 4CH imaging planes.

The reconstruction network showed similar Dice scores to those obtained by 3D CCTA segmentation networks (typically 91–93% for the LA) in the Multi-Modality Whole Heart Segmentation challenge.^15^ Compared to 3D CCTA segmentation, reconstructing 3D label maps from 2D segmentations of 2CH and 4CH views has substantially fewer input features and suffers from misalignment between the two views.

In order to estimate the LA wall area (total LA surface area minus intersections with the LA appendage and pulmonary veins) we predicted 5 labels (background, LA, LV, left and right pulmonary veins, and LA appendage). The predicted LA appendage and pulmonary veins themselves are not as accurate, since the network must learn how to interpolate the training data regions according to features provided by the input data, which was limited to LA and LV labels only. In order to check whether predicting 5 labels results in a degradation of performance for prediction of the LA, we trained another network to predict just the LA 3D structure from the LA and LV slice input. The LA Dice was 93.8% on the 200 CCTA test cases, similar to the 93.7% obtained from the 5-label prediction network (*P* = NS).

*Figure 4* shows worst case predictions for both CCTA and CMR test datasets. In general, performance was degraded if the long axis slices missed the main body of the LA. This was due partly to slice positioning error but also to the typical practice of planning of 2CH and 4CH views to maximise the length of the LV rather than the LA. Kebed *et al.*^16^ noted that planning views to maximize the LA longitudinal dimension can give more accurate results.

### Limitations

The mean absolute errors of the label completion network on the external CMR test dataset were somewhat larger than for the 200 test cases generated from CCTA. This is likely due to different loading conditions between the CMR and CCTA exams (mean of 2.5 months gap between the two scans), and errors in matching a CMR frame to the CCTA. Even with the difficulties listed above, the LA shape reconstruction network shows good results, suggesting the network is able to estimate geometry from two views in the presence of variation in slice positioning and the breath-hold misalignment between views.

This study used manual segmentations of the 2CH and 4CH cine views since the purpose of the study was to validate the reconstruction of 3D geometry rather than the accuracy of the segmentations. However, accurate fully automated methods for segmenting LA and LV from 2CH and 4CH images are becoming available,^17^ and could be combined with the current method to enable fully automated analysis of LA shape and volume at the time of scanning. In-line evaluation on the scanner would give immediate feedback of results, enabling the immediate acquisition of more detailed scans if warranted. Further studies are needed in larger cohorts to determine the prognostic ability of derived values.

## Conclusions

In this study, we developed a deep learning method for estimating the LA volume and surface area by reconstructing the LA shape from 2CH and 4CH CMR image segmentations. Compared with the standard bi-plane area-length method the network showed a much higher accuracy and robustness. Therefore, this method provides a better evaluation of the LA from routine acquisitions, and can be widely deployed in a fully automated pipeline.

## Supplementary material

Supplementary materials are available at *European Heart Journal - Cardiovascular Imaging* online.

## Supplementary Material

jead010_Supplementary_Data

## Data Availability

The code and networks with trained weights will be available from cemrg.org. The data underlying this article were provided by SCOT-HEART investigators by permission. Data will be shared on request to the corresponding author with permission of SCOT-HEART investigators.
