# Supplementary material for: Deep learning estimation of three-dimensional left atrial shape from two-chamber and four-chamber cardiac long axis views
Source: Eur Heart J Cardiovasc Imaging. 2023 Feb 2;24(5):607–15. doi: 10.1093/ehjci/jead010 (PMC10125223; doi:10.1093/ehjci/jead010)
Supplement: jead010_Supplementary_Data [file jead010_supplementary_data.docx]

**Supplemental Material**

We used the shorter length, mean value, and longer length of the two views to calculate the volume and surface area of LA for both traditional and our proposed modified definition of the atrial length defined in Section 2.4. The error (as the ground truth value minus the predicted value) and the absolute error values are shown in the table below.

**Supplemental Table S1. Comparison of traditional and modified atrial length in biplane area-length methods.**

|  |  | LA volume / ml | LA area (total) / cm^2^ |
| --- | --- | --- | --- |
| Signed Error | Traditional – Shorter | -16.3±96.2 | -107.2±1185.9 |
|  | Traditional – Mean | 9.4±22.3 | 29.1±23.6 |
|  | Traditional – Longer | 18.8±18.7 | 38.1±15.2 |
|  | Proposed – Shorter | **7.3±16.4** | **20.7±57.2** |
|  | Proposed – Mean | 8.0±15.9 | 22.8±43.6 |
|  | Proposed - Longer | 8.5±15.6 | 24.4±34.8 |
| Absolute error | Traditional – Shorter | 33.4±91.7 | 149.5±1181.3 |
|  | Traditional – Mean | 18.4±15.6 | 32.8±18.1 |
|  | Traditional – Longer | 20.4±16.9 | 38.1±15.2 |
|  | Proposed – Shorter | **13.0±34.1** | 34.1±50.4 |
|  | Proposed – Mean | **13.0±12.5** | 33.0±36.4 |
|  | Proposed - Longer | **13.0±12.1** | **32.4±27.5** |

Our proposed definition was much more accurate and robust compared to the traditional definition, and the shorter length has the smallest bias for both LA volume and area estimation, while there was no significant difference among the absolute error of the volume estimations using shorter, mean value or longer length of our proposed definition. However, the absolute error calculated using proposed longer length gave the smallest absolute error for LA surface area. We decided to use the shorter length of our proposed definition for area-length method evaluation by considering both the bias and absolute error.

**Supplemental Table S2. Checklist for Artificial Intelligence in Medical Imaging (CLAIM)**

| **Title or Abstract** |  |
| --- | --- |
| 1 Identification as a study of AI methodology, specifying the category of technology used (eg, deep learning) | Title, Abstract Aims |
| 2 Structured summary of study design, methods, results, and conclusions | Abstract |
| **Introduction** |  |
| 3 Scientific and clinical background, including the intended use and clinical role of the AI approach | Introduction Para 1,2,3 |
| 4 Study objectives and hypotheses | Introduction Para 3 |
| **Methods** |  |
| 5 Prospective or retrospective study | Methods Para 1 |
| 6 Study goal, such as model creation, exploratory study, feasibility study, noninferiority trial | Methods Para 1 |
| 7 Data sources | 2.1 Datasets. |
| 8 Eligibility criteria: how, where, and when potentially eligible participants or studies were identified (eg, symptoms, results from previous tests, inclusion in registry, patient-care setting, location, dates) | 2.1 Datasets |
| 9 Data preprocessing steps | 2.2, 2.3 |
| 10 Selection of data subsets, if applicable | 2.2 |
| 11 Definitions of data elements, with references to common data elements | 2.4, 2.6 |
| 12 De-identification methods | 2.1 |
| 13 How missing data were handled | 2.1 |
| 14 Definition of ground truth reference standard, in sufficient detail to allow replication | 2.2, 2.3 |
| 15 Rationale for choosing the reference standard (if alternatives exist) |  |
| 16 Source of ground truth annotations; qualifications and preparation of annotators | 2.2, 2.3 |
| 17 Annotation tools | 2.3 |
| 18 Measurement of inter- and intrarater variability; methods to mitigate variability and/or resolve discrepancies | 2.4 |
| 19 Intended sample size and how it was determined | NA |
| 20 How data were assigned to partitions; specify proportions | 2.2 |
| 21 Level at which partitions are disjoint (eg, image, study, patient, institution) | 2.2 |
| 22 Detailed description of model, including inputs, outputs, all intermediate layers and connections | 2.5 |
| 23 Software libraries, frameworks, and packages | 2.5 |
| 24 Initialization of model parameters (eg, randomization, transfer learning) | 2.5 |
| 25 Details of training approach, including data augmentation, hyperparameters, number of models trained | 2.5 |
| 26 Method of selecting the final model | NA |
| 27 Ensembling techniques, if applicable | NA |
| 28 Metrics of model performance | 2.4; 2.6 |
| 29 Statistical measures of significance and uncertainty (eg, confidence intervals) | 2.7 |
| 30 Robustness or sensitivity analysis | 3.2; 3.3 |
| 32 Validation or testing on external data | 3.3 |
| 31 Methods for explainability or interpretability (eg, saliency maps) and how they were validated | NA |
| **Results** |  |
| 33 Flow of participants or cases, using a diagram to indicate inclusion and exclusion | 2.1; 2.2 |
| 34 Demographic and clinical characteristics of cases in each partition | 2.2 |
| 35 Performance metrics for optimal model(s) on all data partitions | 3.2; 3.3 |
| 36 Estimates of diagnostic accuracy and their precision (such as 95% confidence intervals) | 3.2; 3.3 |
| 37 Failure analysis of incorrectly classified cases | NA |
| 38 Study limitations, including potential bias, statistical uncertainty, and generalizability | 4.3 |
| 39 Implications for practice, including the intended use and/or clinical role | 5 |
| **Other Information** |  |
| 40 Registration number and name of registry | NA |
| 41 Where the full study protocol can be accessed | NA |
| 42 Sources of funding and other support; role of funders | Acknowledgements |
